# Language and social/emotional problems identified at a universal developmental assessment at 30 months

**DOI:** 10.1186/1471-2431-13-206

**Published:** 2013-12-13

**Authors:** Fiona Sim, John O’Dowd, Lucy Thompson, James Law, Susan Macmillan, Michelle Affleck, Christopher Gillberg, Philip Wilson

**Affiliations:** 1 Institute of Health and Wellbeing, College of Medical, Veterinary and Health Sciences, University of Glasgow, Caledonia House, Royal Hospital for Sick Children, Glasgow, Yorkhill, G3 8SJ, UK; 2 Public Health, NHS GG&C, Glasgow, UK; 3 Communication & Language Sciences, University of Newcastle, Newcastle, UK; 4 NHS GG&C, Glasgow, UK; 5 Child & Adolescent Psychiatry, University of Gothenburg, Gothenburg, Sweden; 6 Centre for Rural Health, University of Aberdeen, Centre for Health Sciences, Old Perth Rd, Inverness, IV2 3JH, Scotland

**Keywords:** Child development, Language delay, Socio-emotional development, Screening, Preschool assessment, Child health surveillance, Child psychiatry

## Abstract

**Background:**

Preschool language and neurodevelopmental problems often persist and impede learning. The aims of the current study are to assess the uptake of a new universal 30 month health visitor contact and to quantify the prevalence of language delay and social/emotional difficulties.

**Methods:**

All families of 30 month old children in four Glasgow localities were offered a visit from their health visitor. Structured data were collected relating to language, social and emotional development using three instruments; The Strengths and Difficulties Questionnaire (SDQ), the abbreviated Sure Start Language Measure and a two-item language screen.

**Results:**

From an eligible population of 543 children, there was a 90% return rate of contact forms from the health visitors, and assessments were completed on 78% of eligible children. Visit completion rates did not differ significantly by socio-economic status. 3-8% of children were reported to have language delay depending on the method of assessment. 8.8% of children scored in the “abnormal” range of SDQ total difficulties scores and 31.1% had an abnormality in at least one subscale. There was substantial overlap between language delay and abnormal scores on the SDQ.

**Conclusions:**

Universal assessment of neurodevelopmental function at 30 months identified a significant proportion of children, including those previously considered at low risk, with both language and social/emotional difficulties. Further work is required to assess the precise nature of these difficulties and to assess the potential impact on services.

## Background

Behavioural problems identified in the preschool years often persist [1,2] and their association with adverse physical, mental health and forensic outcomes in adulthood is now generally accepted [2-6]_._

In 2010 the Scottish Government’s Health Department mandated a new universal child health contact between 24-30 months to identify children who might benefit from further support. There had been no universal child health surveillance contact beyond 16 weeks prior to this policy recommendation, though families may have received routine contacts from their health visitor depending on their assessed need and customary practices within their region of the health service. The universal surveillance contact was removed in part because of a lack of evidence demonstrating effectiveness [7].

Language development is closely related to broader social development, and there is a high incidence of language/communication difficulties in children with emotional and behavioural problems [8-12]. The strength of the association between language delays and behaviour problems is however still subject to debate [13].

The majority of late talkers identified in the preschool period tend to move into the normal range by school age [14-18] but continue to have significantly weaker language skills than peers with similar backgrounds. As with behavioural problems, language difficulties identified at this age can persist in some form into later childhood [19-21]_,_ adolescence [22,23] and adult life [24-26]. Although some studies have sought to conduct longitudinal follow-up, much of the research in this area uses retrospective data with older participants from clinical samples and is therefore less representative of the whole population and less informative of the relationship between early language and behavioural development.

There is an increasingly strong case for identification of language and behavioural [27] problems in early childhood given the range of effective interventions for early neurodevelopmental and communication problems [28,29]. Language delay in preschool children is common [30], and many children are not identified until they begin formal education [31]. In the United Kingdom, parents are generally given advice about normal language development and are then expected to identify children with problems and notify their general practitioner or health visitor [32]. The effectiveness of this approach in identifying children with speech and language difficulties is unclear and parental concerns about toddlers predominantly emphasise eating, sleeping and toileting problems [33]. Screening for language delay using standardised measures could be a more reliable method of identifying language and communication problems in the pre-school years [34,35]_._

A pilot evaluation of a universal 30 month contact [36] showed that community child health nurses identified a substantial number of children with language delay and behavioural problems who were previously considered at low risk of developmental difficulties and who would normally not have received any preventative health service input until school entry.

This aims of this study are to a: assess the uptake of a new routine 30 month health visitor contact and b: to quantify the prevalence of language delay and social/emotional/behavioural difficulties and their potential overlap.

## Methods

### Procedure

All families in four regions of Greater Glasgow with children aged 30 months were offered a home visit by their health visitor/public health nurse in August 2011. As well as routine health visiting enquiry, the assessment involved the use of structured instruments assessing social, emotional and behavioural difficulties and language acquisition. The health visitors collected the data on paper forms which were scanned using optical character recognition software for later analysis by the research team at the University of Glasgow. Population denominator data were provided by NHS Greater Glasgow and Clyde.

The area-based Scottish Index of Multiple Deprivation (SIMD) 2009 was used to assign socio-economic status to areas of residence and the Health Plan Indicator (HPI) for Scotland was used to classify previously assessed level of need [37,38]. “Core” HPI status is assigned, usually at 8-16 weeks, to children considered to be at lowest risk. These children do not usually receive any further routine face-to-face health visitor contacts [39]. “Additional” and “Intensive” HPI status is assigned to children considered at higher developmental risk.

### Participants

Families registered with a general practice with children aged 30 months across North East & North West Glasgow, East Dunbartonshire and Renfrewshire were offered a home visit by their health visitor.

### Measures

To assess the development of language and social & emotional skills, the following measures were used:

● The parent report version of the 25-item Strengths and Difficulties Questionnaire [40,41] (SDQ) as the principal measure of social and emotional functioning. Population norms for preschool children were obtained from Prof. Robert Goodman [42] and Dr Adrian Angold [43] based on a population of American 2-5 yr olds. The SDQ contains the following subscales;

o Emotional symptoms

o Conduct problems

o Hyperactivity/inattention

o Peer relationship problems

o Pro-social behaviour

● A four-component language screen including;

● The following questions based on the Law-Miniscalco language screen [44];

■ Does your child use two-word utterances?

■ Can your child say 50 words?

■ Can your child understand 50 words?

o The Sure Start Language Measure, [45] a 50-item word checklist based on the McArthur Communicative Development Inventory [46] the measure which is rapidly becoming the ‘industry standard’ for the description of children’s early expressive language [47]. This was adapted and standardised for use in Sure Start programmes in England [48,49] and a threshold developed for the purposes of early identification.

### Analysis

Data were analysed using descriptive statistics, cross-tabulations and correlation coefficients using IBM SPSS Statistics v19.

Ethical approval was not required for this work as it was conducted as part of a service evaluation. The Caldicott Guardian (data controller) for NHS Greater Glasgow and Clyde gave permission for use of the evaluation data in this report.

## Results

From a potential 543 children in the eligible population for this contact, data were obtained from 486 (90%) children who either received the 30 month contact or had attempted contacts. Full data were obtained on 421 children, 78% of those eligible.

Of the sample of 486 children, 64.8% were assigned to the Core HPI (lowest risk status) before the contact, with 17.5% Additional HPI (intermediate risk) and 6% Intensive HPI (children requiring multi-agency involvement). 10.7% of children had missing HPI information and 1% had not yet had HPI allocated.

Table 1 demonstrates that a large proportion (48.3%) of participants in this sample were, at the time of data collection, living within the most deprived areas of Greater Glasgow.

**Table 1:** Return rates and completed contact rates by socio-economic status

	**SIMD quintile**					
	**1**	**2**	**3**	**4**	**5**	**Total**
Denominator population with valid SIMD data*	262	66	56	65	85	543
N (% of denominator) with contact forms returned	237 (90)	61 (92)	50 (89)	55 (85)	77 (91)	486 (90)
N (% of denominator) completed contacts	206 (79)	53 (80)	44 (79)	47 (72)	71 (84)	421 (78)

Data return rates and visit completion rates by socio-economic status. Scottish Index of Multiple Deprivation (SIMD) quintiles (1 = most deprived, 5 = most affluent) are calculated for postcodes of residence of participants.

*Nine children in the denominator population had missing SIMD data: six were visited and three were not visited.

### Social/emotional/behavioural function (SDQ) data

Table 2 shows values as well as counts of normal (including borderline) or abnormal values for children within each SDQ sub-scale. Provisional cut-off values based on the 90th percentile of a population of American 2-5 yr olds were provided by Dr A. Angold.

**Table 2:** Number and proportion of children in abnormal and normal SDQ groups

**Scale**	**Median (interquartile range)**	**Cut-off scores**	**Normal/ borderline**	**Abnormal**	**Total**
Total	7.5 (5–12)	16	383 (91.2)	37 (8.8)	420
Difficulties					
Emotional	1 (0–2)	3	367 (86.4)	58 (13.6)	425
Symptoms					
Conduct	2 (1–3)	5	368 (86.6)	57 (13.4)	425
Problems					
Hyperactivity	3 (2–5)	7	378 (88.9)	47 (11.1)	425
Peer	1 (0–2)	4	391 (92.0)	34 (8.0)	425
Problems					

There were substantial overlaps between abnormal subscale scores. Of the 151 children who had any subscale abnormality, 78 (52%) had more than one. All children with an abnormal Total Difficulties score had an abnormal score in at least one of the four sub-scales.

### Language data

Table 3 shows that in the brief language screening questions, 3-8% of children had evidence of language delay.

**Table 3:** Language measures

**Language measure**	**Pass**	**Fail**	**Total**
Child says 50 words	389 (92.2%)	33 (7.8%)	422 (100%)
Child puts two words together	412 (96.9%)	13 (3.1%)	425 (100%)
SSLM <10 words	395 (97.3%)	11 (2.7%)	406 (100%)

There were substantial overlaps between problems identified by the different language screening methods. For example, 9/11 (82%) children who were able to say fewer than 10 words from the Sure Start Language Measure (SSLM) were reported to have a total spoken vocabulary of fewer than 50 words. The mean SSLM vocabulary of children reported to use <50 words on the “Can your child say 50 words?” measure was 12 and the mean vocabulary of those reported to use >50 words was 42.

Fourteen Chi-squared analyses were conducted in order to measure the significance of the overlap between language and SDQ results.

Tables 4 and 5 show the overlap between SDQ problems and language delay as assessed by inability to say 50 words and failure to say 10 words from the SSLM list.

**Table 4:** Language measure and abnormal score on SDQ

	**No abnormal SDQ score**	**Any abnormal SDQ score**	**Abnormal total difficulties score**	**Abnormal emotional symptoms score**	**Abnormal conduct problems score**	**Abnormal hyper-activity score**	**Abnormal peer problems score**
Cannot produce 50 words	12/267** (4.5%)	20/149** (13.4%)	8/37** (21.6%)	9/57* (15.8%)	9/56* (16.1%)	7/47* (14.9%)	7/34** (20.6%)
<10 words on SSLM	3/256*	7/144*	1/35	4/55*	0/52	3/47	2/34
	(1.2%)	(4.9%)	(2.9%)	(7.3%)	(0%)	(6.4%)	(5.9%)

(* = P < 0.05, ** = P < 0.01).

**Table 5:** Language measure and abnormal score on SDQ

	**Cannot produce 50 words**	**<10 words on SSLM**
No abnormal SDQ score	12/33 (36.4%) **	3/11 (9.1%) *
Any abnormal SDQ score	20/33 (60.6%) **	7/11 (63.6%) *
Abnormal total difficulties score	8/32 (25%) **	1/10 (10%)
Abnormal emotional symptoms score	9/32 (28.1%) *	4/10 (40%) *
Abnormal conduct problems score	9/32 (28.1%)	0/10 (0%)
Abnormal hyperactivity score	7/32 (21.9%) *	3/10 (30%)
Abnormal peer problems score	7/32 (21.9%) **	2/10 (20%)

(* = P < 0.05, ** = P < 0.01).

### Population distribution of language and social/emotional/behavioural problems

No significant social patterning were identified for either SDQ Total Problems score or for language delay (Chi Square for trend p = 0.26 and 0.82 respectively) (Table 6).

**Table 6:** Gives the distribution of language and SDQ total difficulties scale abnormalities by socio-economic status of area of residence

	**SIMD quintile**					**Total**
	**1**	**2**	**3**	**4**	**5**	
N (%) of denominator population with abnormal SDQ Total Problems Scale score	17 (8.6)	4 (7.4)	2 (4.3)	4 (7.8)	3 (4.3)	30 (7.2)
N (%) of denominator population with vocabulary of <50 words	15 (7.7)	4 (7.4)	1 (2.1)	5 (6.8)	6 (7.9)	31 (7.0)

Sdq Total Problems Scale And Language Delay By Socio-Economic Status. Scottish Index Of Multiple Deprivation (Simd) Quintiles (1 = Most Deprived, 5 = Most Affluent) Are Calculated For Postcodes Of Residence Of Participants.

### Assigned risk status (HPI) and assessed level of difficulty

Overlap quantified using Cross-tabulation and significance tested with Chi-Square analysis (Tables 7 and 8).

**Table 7:** Association between language and HPI status

	**Cannot produce 50 words**	**<10 words on SSLM**
Core HPI	13/33 (39.4%) **	4/11 (36.4%) *
Additional HPI	12/33 (36.4%) **	6/11 (54.5%) *
Intensive HPI	4/33 (12.1%) **	1/11 (9.1%) *

(* = P < 0.05, ** = P < 0.01).

**Table 8:** Association between socio-emotional results and HPI status

	**No Abnormal SDQ score**	**Any abnormal SDQ score**	**Abnormal total difficulties score**	**Abnormal emotional symptoms score**	**Abnormal conduct problems score**	**Abnormal Hyper-activity score**	**Abnormal peer problems score**
Core HPI	193/270 * (71.5%)	89/151 ** (58.9%)	16/37 ** (43.2%)	32/58 * (55.1%)	26/57 ** (45.6%)	24/47 (51.1%)	19/34 (55.9%)
Additional HPI	40/270 * (14.8%)	37/151 ** (24.5%)	14/37 ** (37.8%)	16/58 * (27.6%)	20/57 ** (35.1%)	15/47 (31.9%)	10/34 (29.4%)
Intensive HPI	11/270 * (4.07%)	13/151 ** (8.6%)	5/37 ** (13.5%)	7/58 * (12.1%)	7/57 ** (12.3%)	4/47 (8.5%)	3/34 (8.8%)

(* = P < 0.05, ** = P < 0.01).

Of those children who failed the “Can your child say 50 words?” question, 13 (43%) had previously been allocated to the “core” HPI group (i.e. previously considered to require no routine support) and four (36%) of those failing the SSLM 10 word cut off also had “core” HPI status prior to the 30 month assessment. Of those children who had any abnormal SDQ subscale score, 89 (64%) had previously been allocated to the “core” HPI group.

## Discussion

We sought to assess the uptake of a new routine 30 month health visitor contact and to quantify the prevalence of language delay and social/emotional difficulties.

There was a 90% return rate of contact forms from the health visitors, and assessments were completed on 78% of eligible children. Health visitors were provided with additional local administrative support to allow for time to be allocated to these 30 month contacts. Almost half of the families eligible for assessment had homes in the most deprived quintile of Scottish areas of residence. Contact completion rates did not vary significantly by socio-economic status, which contrasts with earlier Scottish data demonstrating a decline in coverage with increasing age of pre-school children and that this decline was steeper in the most deprived quintile [50].

A large proportion of 30 months old children give indications of having at least some social/emotional or language difficulty. Of the sample of 425 children for whom we have SDQ data; 8.8% were above the threshold for the Total Difficulties Score of the SDQ, while a higher proportion (13.6%) had scores suggesting likely emotional and conduct problems (following further examination of the individual subscales). Similar data have been reported from the Growing up in Scotland cohort at age 34 months [51]. No socio-economic patterning of language or social/emotional/behavioural problems was evident in our sample. This may reflect our modest sample size, but it is equally possible that social gradients demonstrated elsewhere [52] may emerge later in the preschool years.

Based on the simple language screening questions; 3-8% of children in the sample had some indication of language delay.

There was substantial overlap between language delay and social/emotional difficulties as judged by abnormal scores on the SDQ. The association between abnormal SDQ scores and failure to produce 50 words was significant (P < 0.05) for each subscale of the SDQ although the use of multiple comparisons should be noted in interpreting the strength of these associations. Only around one third (12/33) of children with language delay had no abnormal SDQ subscale scores, and children with abnormal SDQ subscale scores are about three times as likely to have language delay than those with no abnormal subscale scores. Our analysis appears to show that it is more common for children who have language delay to also have co-occurring socio-emotional difficulties (60.6% - 63.6%) than for children who have socio-emotional difficulties to also have language delay (13.4% - 4.9%). These findings support other reports that language delay without associated neurodevelopmental problems is relatively uncommon in early childhood [53].

## Conclusions

The 30 month universal nurse contact reported here appears to identify significant developmental needs which may otherwise have gone unmet. We have demonstrated that the assignment of children to the 3 HPI groups in the first 4 months of life based on the anticipated degree of support required did not usefully predict which children would have possible problems at age 30 months. The (English) National Service framework has proposed that decisions about category assignment should be deferred, often to the end of the first year. This view has been was supported by research in health surveillance [54,55] and endorsed in the 2006 revision of Health for All Children.

The pathways from assessment to intervention are currently being optimised and judgement of the overall cost and utility of the 30-month surveillance visit will need to take into account the wider impact on primary care and specialist services. Because screening for communication problems and behavioural difficulties could result in a substantial increase in secondary care workload, it is important to refine any universal screening measures as much as possible in order to identify only those children whose problems are likely to be persistent, to have a serious impact on education and life chances, and be remediable. Further research work is ongoing to ascertain the screening performance of the approach described here, based on parental reporting of difficulties through detailed psychometric assessment of children with and without identified problems. The development of effective interventions to improve outcomes in children with confirmed developmental difficulties at this age remains a high priority.

## Competing interests

The authors declare that they have no competing interests.

## Authors’ contributions

PW, JO’D, CG, JL & LT designed the study, and contributed to the writing of the manuscript. MA conducted statistical analysis of the SIMD data. FS conducted the statistical analysis with contributions from LT and PW and wrote the first draft of the manuscript. All authors read and approved the final manuscript.

## Pre-publication history

The pre-publication history for this paper can be accessed here:

http://www.biomedcentral.com/1471-2431/13/206/prepub
